# Developing food allergy: a potential immunologic pathway linking skin barrier to gut

**DOI:** 10.12688/f1000research.9497.1

**Published:** 2016-11-10

**Authors:** Yui-Hsi Wang

**Affiliations:** 1 Division of Allergy and Immunology, Cincinnati Children's Hospital Medical Center, Cincinnati, OH, 45299-3026, USA

**Keywords:** IgE-mediated food allergy, epidermal TSLP, mucosal mast cells, intestinal mastocytosis

## Abstract

Immunoglobulin E (IgE)-mediated food allergy is an adverse reaction to foods and is driven by uncontrolled type-2 immune responses. Current knowledge cannot explain why only some individuals among those with food allergy are prone to develop life-threatening anaphylaxis. It is increasingly evident that the immunologic mechanisms involved in developing IgE-mediated food allergy are far more complex than allergic sensitization. Clinical observations suggest that patients who develop severe allergic reactions to food are often sensitized through the skin in early infancy. Environmental insults trigger epidermal thymic stromal lymphopoietin and interleukin-33 (IL-33) production, which endows dendritic cells with the ability to induce CD4 ^+^TH2 cell-mediated allergic inflammation. Intestinal IL-25 propagates the allergic immune response by enhancing collaborative interactions between resident type-2 innate lymphoid cells and CD4 ^+^TH2 cells expanded by ingested antigens in the gastrointestinal tract. IL-4 signaling provided by CD4 ^+^TH2 cells induces emigrated mast cell progenitors to become multi-functional IL-9-producing mucosal mast cells, which then expand greatly after repeated food ingestions. Inflammatory cytokine IL-33 promotes the function and maturation of IL-9-producing mucosal mast cells, which amplify intestinal mastocytosis, resulting in increased clinical reactivity to ingested food allergens. These findings provide the plausible view that the combinatorial signals from atopic status, dietary allergen ingestions, and inflammatory cues may govern the perpetuation of allergic reactions from the skin to the gut and promote susceptibility to life-threatening anaphylaxis. Future in-depth studies of the molecular and cellular factors composing these stepwise pathways may facilitate the discovery of biomarkers and therapeutic targets for diagnosing, preventing, and treating food allergy.

## Introduction

Food allergy has emerged as a major health problem worldwide because of the rapid increase in prevalence over the past decade. Food-induced allergic reactions can cause clinical symptoms ranging from mild mouth itching and abdominal pain to life-threatening anaphylaxis, characterized by hypotension, vascular collapse, cardiac dysrhythmias, and diarrhea ^1^. Among the 15 million people who are affected by food allergy in the US, only some individuals develop food-induced, life-threatening anaphylaxis, resulting in 30,000 emergency room visits per year ^2^. In the healthy gastrointestinal (GI) tract, the epithelial lumen and GI immunity develop active immune tolerance to dietary antigens, combat invading microbes, and limit their persistence in the mucosa. It is unclear why some individuals fail to establish oral tolerance toward innocuous food allergens and develop allergic reactions to food allergens at the mucosal sites of the GI tract. Importantly, it is perplexing why only some of the individuals with food allergy who have high levels of dietary allergen-specific serum immunoglobulin E (IgE) acquire susceptibility to developing life-threatening anaphylactic reactions ^3, 4^. Clinically, individuals with atopy and skin sensitization in infancy often develop an allergic response to ingested food in the GI tract later in life ^5– 7^. Although this observation has led to the “dual-allergen exposure” hypothesis, the molecular and cellular mechanisms that support this plausible hypothesis remain to be established. The focus of this review is to discuss recent advances in understanding the molecular and cellular factors that contribute to allergic disease progression and promote susceptibility to life-threatening, IgE-mediated food allergy.

## Epidermal thymic stromal lymphopoietin and interleukin-33 induce allergic sensitization

Recent clinical studies reveal that some patients with atopic dermatitis (AD) in early life may have a higher risk of developing food allergy ^6– 9^. Infants with atopic eczema are prone to be sensitized to egg at only 4 months of age ^10^. In the population-based study of pediatric food allergy, eczema and filaggrin gene loss-of-function mutations, which are associated with reduced skin barrier integrity, are identified as the risk factors for food sensitization ^7^. Evidence from murine studies demonstrates that epicutaneous applications of food proteins can trigger sensitization, which results in the development of IgE-mediated food allergy after repeated food ingestions ^11– 14^. These findings support the notion that the skin barrier is an important route in initiating allergic sensitization to food antigens and evading oral tolerance.

After injury, stress, or environmental insults, the skin epithelium loses its barrier function and orchestrates inflammatory responses and tissue remodeling by producing a myriad of cytokines, chemokines, and growth factors ^15^. Indeed, the idea that skin epithelium can trigger the onset of allergic diseases is supported by the findings that thymic stromal lymphopoietin (TSLP) can endow dendritic cells (DCs) with the ability to create a type 2-permissive microenvironment and drive a T-cell-mediated allergic immune response ^16, 17^. Strong TSLP production is associated with the accumulation of large numbers of DCs activated by DC-lysosome-associated membrane protein-positive and of CD3 ^+^ T cells in the apical layers of the epidermis of patients with AD but not in normal or non-lesional skin ^18^. Mechanistically, keratinocyte-derived TSLP can potently induce the maturation and activation of infiltrated myeloid DCs. These TSLP-activated DCs can produce large amounts of chemokines to recruit inflammatory cells, induce CD4 ^+^ T helper type 2 (TH2) cell differentiation, and maintain functional attributes of CD4 ^+^TH2 memory/effector cells ^18– 20^. In concert with eliciting a DC-mediated TH2 immune response, a recent study showed that TSLP could also promote interleukin-3 (IL-3)-dependent basophil hematopoiesis, resulting in basophil-mediated allergic inflammation ^11, 21^. Perhaps early exposure to food proteins accompanied by environmental insults or genetic predisposition factors that result in epidermal TSLP production may provoke DC/basophil/CD4 ^+^TH2 cell-mediated allergic sensitization via skin barrier before establishing tolerance to ingested foods during infancy.

**Figure 1.**
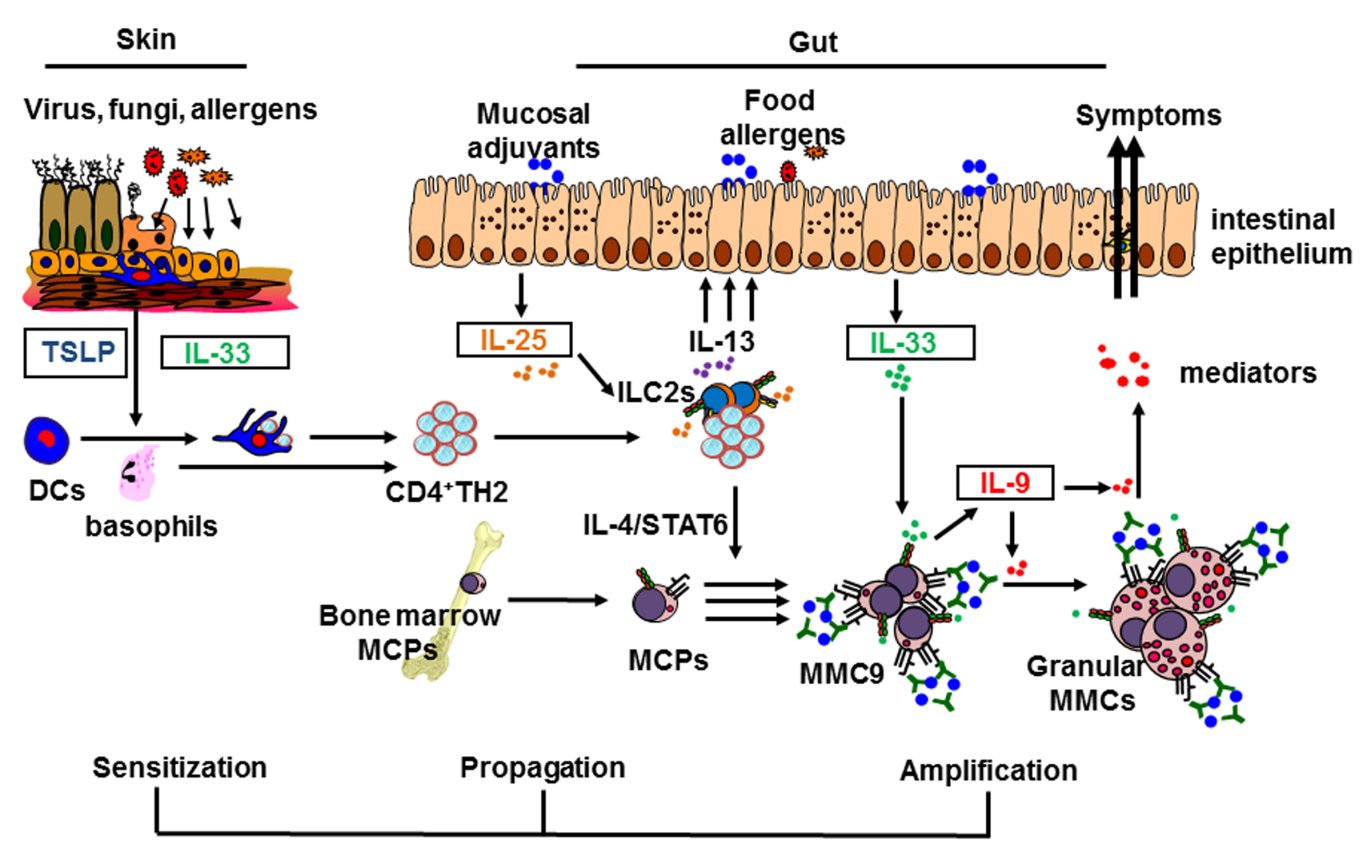
Schematic overview of the stepwise mechanisms involved in the development of immunoglobulin E (IgE)-mediated food allergy. In the allergic sensitization phase, environmental or mechanical triggers (or both) may induce skin keratinocytes to produce thymic stromal lymphopoietin (TSLP), which recruits and activates dendritic cells (DCs) or basophils. Injured epithelial cells may also release interleukin-33 (IL-33) to activate ST2-expressing skin DCs. TSLP-activated DCs migrate to draining lymph nodes to induce naïve CD4 ^+^T cells to differentiate into CD4 ^+^TH2 cells and maintain CD4 ^+^TH2 effector/memory pools. In the allergy propagation phase, these CD4 ^+^TH2 cells migrate to the intestine and interact with resident type-2 innate lymphoid cells (ILC2s) to produce large amounts of IL-13 in response to intestinal IL-25 stimulation. In the amplification-of-mastocytosis phase, IL-4 signals provided by CD4 ^+^TH2 cells induce emigrated mast cell progenitors (MCPs) to become multi-functional IL-9-producing mucosal mast cells (MMC9s), which then expand greatly after ingested antigens cross-link with MMC9 surface IgE/FcεR complex. The inflammatory cytokine IL-33 enhances IL-9 production by MMC9s, resulting in MMC9 maturation and the amplification of intestinal mastocytosis in an autocrine loop. Thus, MMC9 induction may serve as a key cellular checkpoint to amplify and propagate allergic inflammation, resulting in the development of IgE-mediated food allergy. MMC, mucosal mast cell; STAT6, signal transducer and activator of transcription 6; TH2, T helper type 2 cell.

Several factors have been demonstrated to induce epidermal TSLP production. Topical application of vitamin D3 analogs, the ligand for the vitamin D receptor, induces strong TSLP production by keratinocytes, resulting in the development of an AD-like phenotype in mice ^22^. Since the binding of vitamin D receptor and retinoid X receptor alpha (RXRα) or RXRβ heterodimers can form the transcriptional repressor of *Tslp* gene, the treatments of vitamin D3 analogs which alleviate the formation of such transcription repressor result in the induction of *Tslp* gene expression in the mouse skin keratinocytes ^22^. The activation of Toll-like receptors by viral, bacterial, and fungal ligands can also induce TSLP production in epithelial cells ^23^. Accumulating evidence from recent animal studies has further substantiated the roles of TSLP and allergic sensitization via skin barrier in the development of experimental food allergy. Mice that develop AD after repeated topical applications of ovalbumin (OVA) plus vitamin D3 analog lose tolerance to ingested OVA and eventually develop symptoms of experimental food allergy ^11– 13^. Conversely, mice deficient in TSLP receptor specifically in DCs also fail to develop antigen-specific IgE after epicutaneous sensitization and are resistant to developing experimental food allergy ^24^. Overexpression of TSLP can activate intradermally reconstituted basophils to promote cutaneous allergic inflammation, resulting in the development of experimental food allergy ^11^. By contrast, ablation of basophils in mice that are sensitized after topical application of vitamin D3 analogs results in resistance to developing experimental food allergy ^11, 25^. These studies underscore the pivotal role of epidermal TSLP production in orchestrating the DC/basophil-mediated TH2 immune response that initiates the allergic sensitization to food antigens in the skin barrier, leading to the propensity to develop a food allergy ( Figure 1).

In addition to aberrant epidermal TSLP induction, the loss of skin barrier function due to filaggrin gene mutation or injury after repeated skin picking (excoriation) also increases the risk for peanut allergy ^26^. Indeed, direct epicutaneous applications of cashew peanut extract, not soy extract, are sufficient to trigger adjuvant-independent allergic sensitization, possibly mediated by the skin-draining DCs that express ST2, the receptor for the inflammatory cytokine IL-33, also termed alarmin ^14^. In another study, repeated tape stripping, which imitates the excoriation of skin observed in AD patients, can also trigger the production of the epithelial-derived inflammatory cytokine IL-33 and promote the development of experimental food allergy ^27^. Perhaps mechanical skin injury can induce an increase in circulating IL-33, which enhances IgE-mediated mucosal mast cell (MC) degranulation in the gut, resulting in the development of anaphylactic response to ingested antigens ^27^. Thus, in addition to TSLP, the epithelial-derived cytokine IL-33 and the allergenic property of certain food allergens can serve as the alternative factors to induce allergic sensitization to food antigens after skin injury occurred. These findings also broaden our understanding of the factors and immunologic pathways underlying the initiation of skin allergic sensitization that may potentiate the development of food allergy.

## Interleukin-25, type-2 innate lymphoid cells, and CD4 ^+^TH2 cells perpetuate allergic reactions

In addition to allergic sensitization to food antigens via a damaged skin barrier, other factors at the mucosal site of the GI tract may confer susceptibility to food allergy later in life. After the occurrence of allergic sensitization induced by administering epicutaneous TSLP plus OVA antigen, blocking TSLP activity by using anti-TSLP antibody does not prevent skin-sensitized mice from developing experimental food allergy ^24^. This finding implies that the perpetuation and amplification of allergic reactions from skin to small intestine in the GI tract are essential for the development of IgE-mediated food allergy ^24, 25^.

The GI mucosa is the largest immunologic site that constantly encounters numerous varieties of food antigens present in the daily diet. Antigen sampling, processing, and presenting at the mucosal sites of the GI tract are complex processes involving intestinal epithelial cells, M cells, goblet cells, and DCs ^28, 29^. It is postulated that tolerogenic CD103 ^+^ DCs present luminal food antigens to naïve CD4 ^+^ T cells to induce food antigen-specific regulatory CD4 ^+^ T cells, leading to a state of unresponsiveness to ingested antigens or oral tolerance ^30, 31^. Considerable evidence demonstrates that the intestinal epithelial-derived cytokine IL-25 (IL-17E), a distinct IL-17 cytokine member, is a key factor in promoting protective type-2 immunity to parasitic infection ^32, 33^ (for example, helminth) and limits TH1- and TH17-mediated inflammation induced by commensal flora ^34, 35^. Endogenous intestinal IL-25 produced constitutively by tuft cells, one of the five intestinal epithelial cell lineages, can sustain the homeostasis of type-2 innate lymphoid cells (ILC2s) and activate ILC2s to secrete IL-13 after helminth infection ^36^. These studies suggest that intestinal IL-25 may regulate the balance of the immune response to dietary proteins in the GI tract after the occurrence of allergic sensitization. Indeed, allergic sensitization results in the increase of intestinal IL-25 expression, which potentiates the development of allergic reactions to ingested antigens ^37^. Compared with their wild-type controls, genetically modified murine strains that produce intestinal-specific IL-25 constitutively or that lack the IL-25 receptor, IL-17RB, are more susceptible or resistant, respectively, to developing IgE-mediated experimental food allergy ^37^. Although intestinal ILC2s are the primary TH2 cytokine producers in response to IL-25 stimulation, ILC2s alone are insufficient to drive anaphylactic reactions to ingested antigens in naïve or sensitized transgenic mice that produce IL-25 constitutively ^37^. Notably, CD4 ^+^TH2 cells that are induced after allergic sensitization and then amplified after repeated ingested antigen challenge are required for ILC2s to produce large amounts of IL-5 and IL-13 in response to IL-25 stimulation, resulting in the development of experimental food allergy ^37^. Possibly, IL-2 production by ingested antigen-induced CD4 ^+^TH2 cells promotes the capabilities of ILC2s to produce IL-5 and IL-13 in response to intestinal IL-25 stimulation ^37– 40^. These findings may also explain the observation that IL-25-deficient mice infected with *Trichuris muris*, a GI parasite, fail to develop lymphocyte-dependent protective type-2 immunity to expel chronic parasitic infection ^33^. In another study using mice expressing a gain-of-function mutation of IL-4 receptor α chain ( *Il4raF709*), ILC2s are found to produce some IL-4 in response to IL-33 stimulation in a mouse model of food allergy sensitized with staphylococcal enterotoxin B ^41^. ILC2-derived IL-4 promotes the development of experimental food allergy by dampening regulatory T cell function, which can directly suppress mucosal MC function ^41^. Together, these studies substantiate the role of IL-25 in promoting intestinal allergic reaction to ingested antigens by enhancing the concerted interactions between ILC2s, antigen-induced CD4 ^+^TH2 cells, or regulatory T cells (or a combination of these) after the occurrence of allergic sensitization ^25^. Furthermore, the findings support the view that IL-25 may bridge the crosstalk between the skin and gut by mediating collaborative interactions between ILC2s and CD4 ^+^TH2 cells to amplify the cascade of allergic reactions to ingested antigens at the effector phase of IgE-mediated food allergy ( Figure 1).

## Interleukin-33 and type-2 mucosal mast cells amplify hypersensitivity reactions

Food-induced anaphylaxis is an immediate, adverse reaction triggered predominantly by cross-linking of antigen-specific IgE bound to the high-affinity IgE receptor FcεR on MCs after re-exposure to allergen ^42– 44^. Mechanistically, FcεR cross-linking activates a downstream signaling cascade that causes rapid release of vasoactive and preformed mediators, including histamine, tryptase, carboxypeptidase A, leukotrienes, and platelet-activating factor, resulting in physiological alternations that cause shock (anaphylaxis) ^45, 46^. However, it is perplexing why only some, rather than all, individuals who are competent to generate MCs and have high levels of dietary allergen-specific IgE develop life-threatening anaphylaxis ^3, 4^. Similar to humans, sensitized murine strains that have normal MC development and acquire high amounts of antigen-specific IgE vary in their susceptibility to developing severe systemic anaphylaxis ^13^. This enigma hints that in addition to IgE and MCs, other molecular and cellular factors may also participate in driving the development of life-threatening anaphylaxis. Considerable evidence from clinical and animal studies demonstrates that elevated levels of IgE-positive MCs in the small intestine are associated with food allergy ^42, 47– 49^, suggesting a pivotal role of GI MCs in the development of food-induced, life-threatening anaphylaxis ^43, 50^. Taking advantage of the differences in their susceptibility to food allergy, comparative analyses of intestinal mucosal cellular components among examined murine strains led to the identification of the novel, multi-functional IL-9-producing mucosal MCs (MMC9s) ^13^. MMC9s function as type-2-promoting innate myeloid cells by producing prodigious amounts of the TH2 cytokines IL-9 and IL-13 and exert MC function by secreting histamine and MC proteases ^13^. Unlike conventional MCs, MMC9s display innate helper cell-like morphology with few metachromatic granules in their scanty cytoplasm. It appears that MMC9s are scarce in the small intestines of immunologically naïve mice. After allergic sensitization via the skin barrier or TH2-promoting adjuvant, the atopic IL-4 signaling provided by ingested antigen-induced CD4 ^+^TH2 cells can induce FcεR-expressing MC progenitors from bone marrow to develop into MMC9s ^13^. Thus, mice expressing a gain-of-function mutation of IL-4 receptor α chain ( *Il4raF709*) are more prone to developing experimental food allergy because of a cell-intrinsic effect of IL-4 in intestinal MC homeostasis ^51^. Furthermore, MMC9s expand greatly in the small intestine of wild-type mice, but not mice deficient in FcεRα, after repeated exposure to ingested antigens, indicating that cross-linking of the surface IgE/FcεR complex by ingested antigens promotes the proliferation of MMC9s ^13^. Reciprocally, ingested antigen-induced MMC9s amplify CD4 ^+^TH2 cell immune responses, which drive the concomitant increase of MMC9 and CD4 ^+^TH2 cell occurrence (unpublished observation). Furthermore, robust IL-9 production by MMC9s is essential for effective MMC9 expansion, which promotes intestinal mastocytosis in an IL-9-dependent autocrine manner, resulting in the development of severe systemic anaphylaxis ^13^. Notably, intestinal epithelial-derived IL-33 can enhance the function of MMC9s by inducing robust IL-9 production, which can amplify intestinal mastocytosis, resulting in the development of anaphylactic response to ingested antigens ^13^. It appears that in addition to promoting allergic reactions to ingested foods, intestinal MCs can provide an IL-4 signal to induce a TH2 cell program in regulatory T cells, resulting in the impairment of regulatory T-cell function and the loss of tolerance ^52^. Given their anatomical location, characteristics, and function, MMC9s may be a key player that bridges the crosstalk between the skin and gut by perpetuating allergic reactions and amplifying anaphylactic responses to dietary proteins. Indeed, among sensitized murine strains, MMC9 occurrence is positively associated with their susceptibility to experimental food allergy ^13^. Ablating MMC9s results in resistance to developing experimental food allergy ^13^. In human studies, increased duodenal MMC9 frequency and expression levels of *Il9* and MC-specific transcripts are associated with atopy patients who developed comorbid allergic diseases, such as eczema and food allergy ^13^. Thus, MMC9 induction may represent a pivotal cellular checkpoint in acquiring susceptibility to developing life-threatening anaphylaxis. Furthermore, these findings represent a new conceptual paradigm by linking atopic status (IL-4), dietary antigen and IgE/FcεR complex interactions, and inflammatory cues (exemplified by IL-33) with MMC9 biology and food allergy ( Figure 1).

## Conclusions

Although our knowledge of the pathways underpinning the development of allergy has increased, current evidence does not yet fully explain why life-threatening anaphylaxis occurs in only some individuals among those who are allergic to food allergens. Over the past decade, considerable evidence has led to a plausible hypothesis that details the stepwise mechanisms involved in the development of food allergy (illustrated in Figure 1): (i) in the allergic sensitization phase, exogenous molecules (lectins, proteases, or chitins) acting as mucosal TH2 adjuvants and filaggrin as a genetic predisposing factor may initiate inflammatory reactions to induce the production of epidermal TSLP or IL-33 (or both) that triggers allergic sensitization to contacted food allergens before the establishment of oral tolerance ^53– 56^. TSLP endows DCs and potentiates basophil function to promote a TH2-permissive microenvironment, which induces CD4 ^+^TH2 cells to differentiate and maintain antigen-specific CD4 ^+^TH2 memory/effector cells ^16, 18, 19, 57^, which migrate to a draining lymphoid node and induce antigen-specific IgE generation. (ii) In the allergy propagation phase, re-exposure to ingested food antigens activates emigrated antigen-specific CD4 ^+^TH2 memory/effector cells in the small intestine to produce IL-13, resulting in the increase of intestinal IL-25 production. In the presence of CD4 ^+^TH2 memory/effector cells and IL-25 stimulation, resident ILC2s produce large amounts of IL-13, which creates a TH2-permissive environment that prevents tolerance. (iii) In the amplification-of-mastocytosis phase, repeated food antigen ingestion induces the increase of CD4 ^+^TH2 cells, which provide the IL-4 signaling that induces MC progenitors to develop into MMC9s, which expand greatly after ingested antigens cross-link with MMC9 surface IgE/FcεR complex. IL-33, a potent inflammatory cytokine, enhances IL-9 production by MMC9s, resulting in MMC9 maturation and the amplification of intestinal mastocytosis in an autocrine loop. Thus, it is possible that local accumulation of IgE-bearing MMC9s will impose the potent reactivity to ingested food antigens and is the prerequisite for developing life-threatening anaphylaxis. The proposed model provides paradigm-shifting insight into the immunologic mechanisms composing the progression of allergic reactions to food allergens from the skin to gut. Indeed, our proposed notion mirrors recent changes in the guidelines of the American Academy of Pediatrics, including the recommendation for early oral exposure to food allergens during infancy for children at risk of atopic diseases ^58^. Future in-depth studies of human MMC9 and MMC9-associated molecules involved in the clinical reactivity of food allergy will provide the basis to translate the mechanistic findings from murine studies to further our understanding of human food allergy and the clinical application of this knowledge.

## Abbreviations

AD, atopic dermatitis; DC, dendritic cell; GI, gastrointestinal; IgE, immunoglobulin E; IL, interleukin; ILC2, type-2 innate lymphoid cell; MC, mast cell; MMC9, interleukin-9-producing mucosal mast cell; OVA, ovalbumin; RXR, retinoid X receptor; TH2, T helper type 2; TSLP, thymic stromal lymphopoietin.
